# Immune-mediated liver injury of the cancer therapeutic antibody catumaxomab targeting EpCAM, CD3 and Fcγ receptors

**DOI:** 10.18632/oncotarget.8574

**Published:** 2016-04-04

**Authors:** Jürgen Borlak, Florian Länger, Reinhard Spanel, Georg Schöndorfer, Christian Dittrich

**Affiliations:** ^1^ Centre for Pharmacology and Toxicology, Hannover Medical School, Hannover, Germany; ^2^ Department of Pathology, Hannover Medical School, Hannover, Germany; ^3^ Institute of Pathology, Viersen, Germany; ^4^ Clinical Development, Fresenius Biotech GmbH, München, Germany; ^5^ Applied Cancer Research – Institution for Translational Research Vienna (ACR-ITR VIEnna) and Ludwig Boltzmann Institute for Applied Cancer Research (LBI-ACR VIEnna), Center for Oncology and Hematology, Kaiser Franz Josef-Spital, Vienna, Austria

**Keywords:** catumaxomab, acute liver failure (ALF), idiosyncratic drug hepatotoxicity, epithelial cell adhesion molecule EpCAM

## Abstract

The immunotherapeutic catumaxomab targets EpCAM positive cancers and is approved for the treatment of peritoneal carcinomatosis. To assess the safety of intravenous applications a phase 1 clinical trial was initiated. Treatment of EpCAM positive tumor patients with catumaxomab caused dose dependent hepatitis as evidenced by significant elevations in serum alanine- and aspartate aminotransferases, bilirubin, γGT and induction of the acute phase C-reactive protein (CRP) and the cytokines IL6 and IL8. The first patient receiving 10μg catumaxomab experienced fatal acute liver failure which led to the termination of the study. Immmunopathology revealed catumaxomab to bind via its Fc-fragment to FcγR-positive Kupffer cells to stimulate CRP, chemokine and cytokine release. The observed CD3+T-cell margination at activated hepatic macrophages exacerbated T-cell mediated cytotoxicity. Strikingly, the combined Kupffer/T-cell responses against liver cells did not require hepatocytes to be EpCAM-positive. Catumaxomab's off-target activity involved T-cell mediated lysis of the granzyme B cell death pathway and the molecular interaction of hepatic sinusoidal macrophages with T-cells induced cytolytic hepatitis. Although the bile ducts were surrounded by densely packed lymphocytes these rarely infiltrated the ducts to suggest an intrahepatic cholestasis as the cause of hyperbilirubinaemia. Lastly, evidence for the programming of memory T-cells was observed with one patient that succumbed to his cancer six weeks after the last catumaxomab infusion. In conclusion, our study exemplifies off-target hepatotoxicity with molecularly targeted therapy and highlights the complexities in the clinical development of immunotherapeutic antibodies.

## INTRODUCTION

In 2009 the immunotherapeutic antibody Catumaxomab (Removab) was approved for the treatment of peritoneal carcinomatosis by the European Medicine Agency. This bi-specific (antiEpCAM & antiCD3) trifunctional (FCγ Receptors) antibody binds to the epithelial cell adhesion molecule EpCAM, to CD3^+^T-cells and to macrophages as well as dendritic- and NK cells via Fcγ receptors and leads to an MHC unrestricted killing of EpCAM positive tumor cells without co-stimulation [1]. The interaction of different immune effector cells at the tumor site results in complex immune reactions with transient and primarily local cytokine release, antibody-dependent cell-mediated cytotoxicity (ADCC) and phagocytosis of tumor cells.

In order to evaluate the safety and tolerability of ascending intravenous doses of catumaxomab in patients with chemotherapy refractory EpCAM positive cancers a phase I, open label, dose escalating trial was carried out [2]. The study revealed dose dependent hepatotoxicity of different grades with one patient experiencing fulminant fatal acute liver failure (ALF) which led to the termination of the study. In pursue of mechanisms a range of liver function tests and cytokines in the systemic regulation were evaluated and complemented by comprehensive histopathology and immunohistochemistry studies as to inform on the pathomechanism involved.

## RESULTS

**Figure 1:**
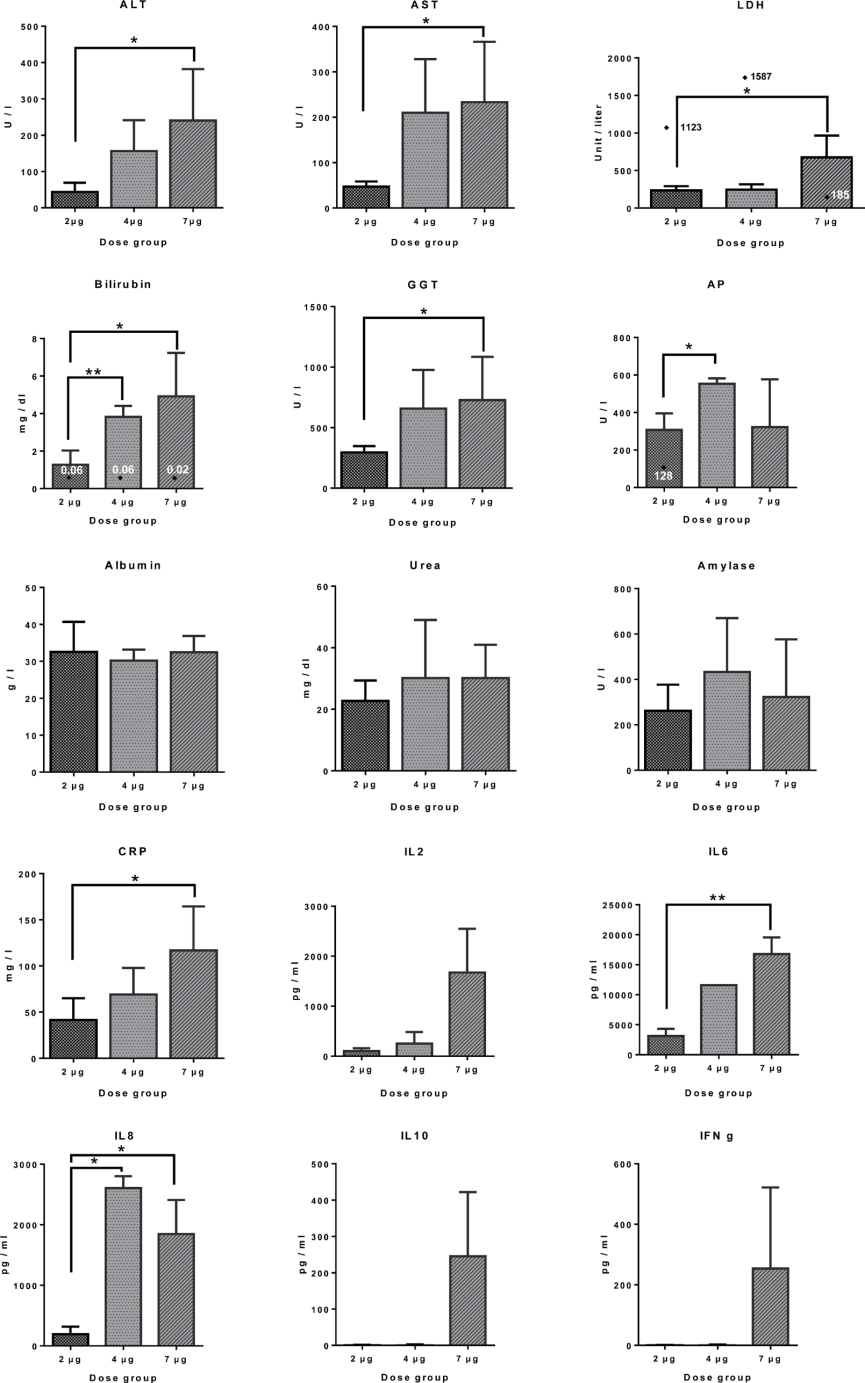
Clinical liver function tests and serum cytokine profiling in patients receiving different doses of catumaxomab infusions Serum liver function tests were assayed 24h after the 2nd catumaxomb infusion, while those for cytokines were assayed 6h after the first infusion, i.e. peak concentrations. Data are given as mean and standard deviation and the statistical significance was determined with the GraphPad software Prism version 6.0. Individual outliers are marked by a diamond symbol. * p<0.02 ** p<0.01.

The findings of a Phase 1 trial in patients with chemotherapy refractory EpCAM positive cancers were recently published [2]. As shown in Figure 1 the transaminases ALT, AST as well as bilirubin and γGT increased dose dependently when assayed 24h after the 2^nd^ catumaxomab infusion, however, alkaline phosphatase, albumin and urea was unchanged. Alike, C-reactive protein was dose dependently increased as was Il-6 and IL-8 while the other cytokines, i.e. IL2, IL10 and interferon gamma did not reach statistical significance when compared to pre-dose values 6h after the first infusion. In the case of lactate dehydrogenase one patient each of the 2μg and 4μg dose had extraordinary high LDH values, and these are marked individually in Figure 1. Likewise for bilirubin there was one patient each per dose group with extremely low laboratory values, i.e. up to 250-fold less when compared to the mean of the dose group. Once again, these are marked individually as a diamond symbol in Figure 1.

Most patients at the 4 and 7μg dose developed anti-drug-antibodies (ADA) after the 4^th^ catumaxomab infusion, and the occurrence of ADA coincided with a significant improvement of the observed hepatitis. Nonetheless, the first patient receiving the 10μg catumaxomab infusion experienced fulminant fatal ALF, and the epicrisis is given below.

### A case of fatal acute liver failure

A 49 year old female patient diagnosed with stage IV colorectal cancer (CRC) and multiple lung but no liver metastasis was scheduled to receive a 10μg catumaxomab infusion. The patient history prior to the study therapy involved three lines of systemic therapies after status hemicolectomy for the treatment of advanced CRC and consisted of 5-fluorouracil (5-FU), folinic acid (FA), capecitabene, oxaliplatin, irinotecan and bevacizumab. The first-line regimen (from 11/2009 to 5/2010) comprised capecitabine/oxaliplatin and bevacizumab, the second-line therapy (from 10/2010 to 11/2010) involved FUFA and irinotecan. The third-line therapy (from 12/2010 to 1/2012) consisted of FUFA and irinotecan in combination with bevacizumab. Only the first-line regimen led to objective remission of lung metastases, and histopathology confirmed lung lesions to be colon cancer derived metastases.

Before study admission the clinical laboratory values were mostly within normal range although a few parameters were elevated to protocol compatible CTCAE Grade 1 (Table 1). Specifically, under the 3rd line chemotherapy, liver function tests were increased to CTCAE Grade 1 and remained increased during therapy. At regular intervals the performed computed tomography evidenced progressive disease in form of an increased seize of several lung metastases but also revealed hepatic steatosis presumably due to the patient's higher BMI score of 36.7.

**Table 1:** Time course of laboratory values prior to and after catumaxomab infusion in a patient with acute fatal liver failure

*10μg CAT IV on 10-Apr-2012 from 10.36h to 16.45h*	prior to IV CAT infusion			after IV CAT infusion					
Parameter (normal ranges) / date	*02-Jan-2012*	*04-Apr-2012*	*10-Apr-2012, 8.05h*	*10-Apr-2012, 18.35h*	*11-Apr-2012, 2:05h*	*11-Apr-2012, 4:25h*	*11-Apr-2012, 6:50h*	*11-Apr-2012, 7:20h*	*11-Apr-2012, 16:50h*
**leukocytes (4.00-10.00G/L)**	**11.18**	6.96	6.58	**2.51**	**10.54**		**18.78**	**19.80**	**19.3**
**hemoglobin** **(12.5-16.00g/dL)**	12.9	13.2	12.7	**11.5**	**9.6**		**10.3**	**10.2**	**8.1**
**haematokrit** **(38.0-44.0%)**	**37.6**	38.1	**37.3**	**33.5**					**23.9**
**thrombocytes** **(150-350 G./L)**	203	264	259	**32**	**30**		**56**	**48**	**46**
**sodium** **(136-145 mmol/L)**	138	138	136	141	141		142	143	**152**
**potassium** **(3.4-4.5 mmol/L)**	4.0	3.7	3.7	**5.0**	**5.0**		**5.2**	**5.1**	4
**calcium** **(2.15-2.50 mmol/L)**	2.31	2.40	2.23	2.15	**1.94**		**1.85**	**1.87**	
**magnesium** **(0.7-1.0 mmol/L)**		0.8	0.8	**0.6**				1.0	
**chloride** **(95-110 mmol/L)**		100	101	104	106		103	100	97
**phosphate** **(0.81-1.45 mmol/L**	0.95	0.95	0.96	**0.53**	**3.08**		**3.59**	**3.62**	**3.9**
**iron** **(33-193 mcg/dL)**		85	93	59					
**BUN** **(6-25 mg/dL)**	16	10	9	10	11		11	12	10
**creatinine** **(0.5-1.0 mg/dL)**	0.59	0.71	0.69	0.95	**1.87**		**2.36**	**2.40**	**2.65**
**GFR/1.7m2BS (MDRD) (90-)**									
**uric acid** **(2.5-6.0 mg/dL)**	5.0	6.2	5.6	6.5	**8.4**		**9.5**	**9.2**	
**glucose** **(50-100 mg/dL)**	**120**	**166**	**148**	100	75		**140**	**139**	
**triglycerides** **(50-150 mg/dL)**			**495**					**383**	
**cholesterin** **(150-200 mg/dL)**			**285**					**4770**	
**Chol/HDL-chol ratio (0-3.6)**			**9.2**						
**bilirubin** **(0.0-1.2 mg/dL)**	0.3	0.35	0.35	1.2	**1.42**		**1.85**	**1.85**	**1.33**
**ammonia** **(11.0-51.0 mcg/L)**						**252.6**	**250.4**		
**lipase** **(13-60U/L)**	32	33		35	**115**		**528**	**518**	**572**
**AP** **(35-105U/L)**	79	93	86	**124**	103		**129**	**121**	**175**
**ASAT (GOT)** **(0-35u/L)**	28	**43**	**37**	**492**	**1759**		**5656**		**13 322**
**ALAT (GPT)** **(0-35U/L)**	35	**56**	**51**	**394**	**1717**		**5554**	**5744**	**9 509**
**Gamma-GT** **(0-40U/L)**	**61**	**52**	**59**	**375**				**225**	
**LDH** **(135-214U/L)**	187	**219**	200	**721**	**2767**		**9476**		**15 500**
**CK** **(20-180U/L)**		135			129		**283**	**284**	**2035**
**total protein** **(66-87g/L)**	67	75	73	**58**	**40**		**42**	**42**	**37**
**albumin** **(35-52g/L)**		39.30	36.6	**29.9**					
**CRP** **(<5 mg/L)**	**55.4**	**17.4**	**14.9**	**19.3**	**24.1**		**27.2**	**26.3**	**15.9**
**NT-pro-BNP** **(0-153 ng/L)**				**207**					
**ferritin** **(50-150 mcg/L)**			**182**						
**PTT** **(70-130%)**	95.3	121		**52.6**	**12.9**		**<10.0**	**<10.0**	**21.6**
**INR**	1.03	<1.00		**1.40**	**5.41**		**>6.0**	**>6.0**	
**aPTT** **(26.0-38.0sec)**		26.1		35.2	**>120**		**>120**	**>120**	**>120**
**fibrinogen** **(1.6-4.0g/L)**		**4.5**		2.1	**<0.5**		**<0.5**		
**D-dimer** **(0.0-0.5 mcg/L)**					**29.01**				
**AT III activity (74.0-126.0%)**		89.9		**62.4**	**23.8**		**22.3**		
**cholinesterase (5320-12920U/L)**	7456	8506		7083					
**α-fetoprotein (0-7.0 mcg/L)**		2.86		1.70					
**CEA** **(0-5.2 mcg/L)**	**6.82**	**9.54**	**10.08**	**21.06**					
**CA 19-9** **(0-34kU/L)**	21.24	31.22	30.04	24.43					
**arterial blood gas analysis**				***23:30h***					
**pH (7.35-7.45)**				**6.85**					
**pCO2 (32-43 mmHg)**				34					
**pO2 (72-100 mmHg)**				87 mmHg					
**lactate**				**>15**					
**HCO3- (23-27 mmol/L)**				**6.1**					
**TCO2**				7.1					
**BEecf (−2-+3 mmol/L)**				**-27.4**					
**BE(B)**				**-26.6**					
**SO2 (95-99%)**				**85**					

Differential blood count and urinalysis prior to catumaxomab infusion was within normal ranges. Equally, thyroid hormone values were within normal ranges.

**Serology 04-Apr-2012:**

HAV-IgM: negative

HAV-IgG: **positive**

HBV surface antigen: negative

HBV core antigen: negative

HCV: negative

HIV: negative

The patient was admitted to the Kaiser-Franz-Josef Spital of Vienna, Austria to obtain an experimental therapy in the frame of a phase I clinical trial of intravenous catumaxomab and was the first to receive the dosage of 10μg of catumaxomab as 6-hour infusion under premedication with 1g of paracetamol to suppress treatment related chill symptoms. The general health condition of the patient was considered to be good but during the catumaxomab infusion the patient started to chill and developed fever (up to 38.9°C). This was not unexpected and was commonly observed amongst patients receiving catumaxomab. In compliance with the study protocol the NSAID ketoprofen was administered at a dose of 50 mg i.v. as a short-term infusion at two consecutive instances one hour apart. Subsequently, the symptoms improved, however, within the next hour a significant decrease in blood pressure (106/55 mmHg) was noted; therefore an additional 1000 mL NaCl was infused via the port-a-cath. At the end of the catumaxomab infusion a further 1 gram of paracetamol was given intravenously as foreseen in the protocol. About 1.5h after termination of the catumaxomab infusion the patient experienced nausea and vomiting (CTCAE Grade 1) in addition to CTCAE Grade 2 vertigo. The patient was given 8 mg of ondansetron for the treatment of nausea and vomiting as well as 1000 mL lactate Ringer solution to treat symptoms of vertigo. Two hours post catumaxomab infusion laboratory values for leukocytes, thrombocytes and albumin were decreased but increased for liver function and coagulation parameters (Table 1). After an additional 30 minutes the patient experienced aggravating upper abdominal pain and therefore received 10 mg morphine hydrochloride. Five hours post catumaxomab infusion the abdominal pain got worse despite the various analgesic medications and due to the deteriorating condition of the patient a mesenteric infarction was suspected. Therefore, an abdominal CT-scan was performed. This revealed a faintly contrasted liver and portal vein with fluid retention around the duodenal knee and partially around the pancreas. The hepatic segment of the inferior vena cava appeared flattened, and the hepatic veins were sparsely calibrated and weakly contrasted. Notwithstanding, the arterial vessels, particularly the mesenteric vessels were without pathological findings. The condition of the patient continued to worsen with a severe drop in blood pressure (81/52 mmHg), blood gas values of pO2 of 87 mmHg, arterial O2 of 85% and alarming changes in blood lactate, pH and coagulation parameters, i.e. >15 mmol/L, pH 6.86 and INR>5, respectively and required intensive care submission. The patient became increasingly unstable in her hemodynamic condition with obligatory high dose noradrenaline treatment while the metabolic acidosis remained unimproved. Approximately 24h post infusion of catumaxomab the patient died from fulminant acute liver failure (ALF); the performed autopsy confirmed ALF as the cause of death and a causal relationship to catumaxomab treatment was considered to be certain.

### Clinical laboratory findings

Changes of several liver function tests, i.e. AST, ALT and albumin were highly significantly regulated and associated with those of LDH and cholesterol (released from membranes due to massive cell damage) indicating massive acute cytolytic hepatitis. Importantly, the cytokine mediated imbalance between coagulant and anticoagulant adjust components of the coagulation system. Inflammation induced activation of coagulation is counteracted by several mechanisms and includes regulation of soluble inhibitors and tissue factor pathway inhibitors (TFPI). Approximately 9 hours post infusion an INR>5, a highly significant increase in D-dimers (> 58-fold) and a highly significant decrease in fibrinogen to < 30% were observed thus evidencing an “overshooting” of the anticoagulant pathway.

### Cytokine release and innate immunity response to catumaxomab

When compared to maxima seen after 2 and 4μg catumaxomab infusions the deceased patient had 15- and 5-fold higher serum IL-6 concentration; nonetheless it did not differ to peak IL-6 serum concentrations after infusion of 7μg catumaxomab as determined in a phase I trial [2]. Importantly, IL-6 plays a decisive role in liver regeneration and was shown to enhance cell survival through activation of hepatoprotective pathways [3]. Conversely, peak serum IL-8 concentration was only about 40% of peak concentrations of the 7μg catumaxomab dose. This cytokine is elevated in serum of patients with chronic liver disease and is known to stimulate activation of neutrophils, macrophages and T-cells via interaction with CXCR1 and CXCR2 therefore contributing to hepatic inflammation [4]. The reasonable good relationship between IL-6 and IL-8 serum levels amongst patients receiving different catumaxomab doses is suggestive for a coordinated dose dependent immune response.

A further finding was the significant rise in TNFα. When compared with the 2, 4 and 7μg catumaxomab doses the peak serum TNFα levels were 7-, 34- and 1.6-fold increased at the end of the infusion. The deceased patient had the highest TNFα serum concentration measured so far and ssensitization of hepatocytes to TNFα and other death receptor pathways imposes high risk for liver injury. As shown in Table 2 several pro- and anti-inflammatory cytokines were significantly elevated with possible spilling from the liver into systemic circulation to perpetuate ALF.

**Table 2:** Cytokine serum concentrations after catumaxomab infusions

Patient	7μg Catumaxomab iv Patient AT0106			10μg Catumaxomab iv Patient AT0107	
	Start of the infusion	1. infusion, 6hrs	2. infusion, 6hrs	Start of the infusion	1. infusion, 6hrs
Catumaxomab [ng/mL]	<LLOQ	0,25	0,2	0,24	0,62
ADA [ng/]	<13	<13	<13	<13	<13
IL-2 [pg/mL]	<LLOQ	2.217,4	491,5	<LLOQ	980,6
IL-4 [pg/mL]	<LLOQ	<LLOQ	<LLOQ	<LLOQ	<LLOQ
IL-6 [pg/mL]	<LLOQ	35.408,0	37.051,2	<LLOQ	59.806,7
IL-8 [pg/mL]	71,76	13.380,3	14.069,8	<LLOQ	5.387,8
IL-10 [pg/mL]	<LLOQ	430,3	5.386,1	<LLOQ	408,7
IL-12p70 [pg/mL]	<LLOQ	<LLOQ	<LLOQ	nd	nd
IFN-g [pg/mL]	<LLOQ	2.077,3	1.715,7	<LLOQ	1.797,6
GM-CSF [pg/mL]	<LLOQ	124,1	<LLOQ	<LLOQ	93,2
TNF [pg/mL]	<LLOQ	850,3	504,8	<LLOQ	1.353,6
CRP [mg/l]	6,7	9,8	12,5	14,9	24,1

Owing to its molecular functions we hypothesized a direct interaction of catumaxomab with hepatocytes. Strikingly, the patients hepatocytes were EpCAM negative (see histopathology/immunohistochemistry section). Therefore, alternative causes of this case of fatal ALF were explored as detailed below.

### Catumaxomab induced sterile inflammation

Research into acute liver failure is highly suggestive for harmed hepatocytes to release damage associated molecular pattern molecules (DAMPs) [5]. These interact with DAMP receptor of sensing immune cells via Toll like receptors such as TLR-4 and release inflammatory cytokines as part of programmed “sterile inflammation” [6]. Several molecular interaction partners of DAMPs are known and include heat shock protein 70, uric acid, ATP, nucleic acids as well as high-mobility group box-1 protein (HMGB1). Once released by damaged cells HMGB1 acts as danger molecule and triggers the inflammatory signalling cascade. Current research suggests posttranslational modifications such as oxidation and acetylation to modulate the pro-inflammatory potential of HMGB1 as danger signal [7]. Furthermore, a recent study showed HMGB1 to be significantly elevated in colorectal cancer patients [8] to possibly influence the risk for sterile inflammation and hence idiosyncratic hepatotoxicity.

**Figure 2:**
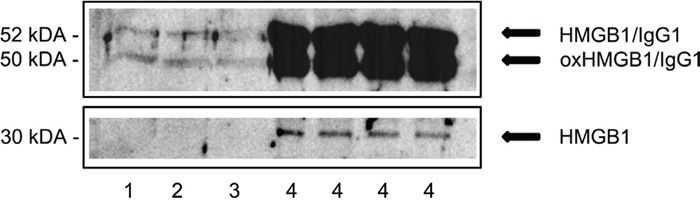
Western Blotting of serum HMGB1 # **1-3.** Depicted are immunoblots of HMGB1 of three healthy individuals. # **4.** Depicted is the immunoblot of HMGB1 of a female patient with fulminant fatal acute liver failure at catumaxomab Cmax concentration. Note, shown are repeats of the same serum sample that was taken at the end of the infusion, i.e. 6h (tmax).

To probe for serum and tissue HMGB1 expression both Western Blotting and immunohistochemistry was performed. As depicted in Figure 2 marked serum expression of HMGB1 was observed. Next to the expected 30 kDa immunoreactive band two additional bands at a molecular weight of about 50 and 52 kDa were observed. Based on the molecular weight of HMGB1 (30kDa) and the molecular weight of the light chain of IgG1 (22kDa) binding of IgG1 to HMGB1 is assumed. Specifically, the binding of HMGB1 to several proteins including IgG1 was confirmed by co-immunoprecipitation of serum proteins with anti-HMGB1 antibodies [9]. Depletion of albumin and IgG from serum confirmed binding of HMGB1 to immunoglobulin. A further important finding was the significant increase in serum uric acid (Table 1). Although not confirmed by electron microscopy it was established that crystals of uric acid function as second signal required for an activation of the inflammasome and downstream caspases to reinforce cellular damage and programmed cell death programs. Taken collectively, HMGB1 and serum uric acid were significantly elevated. We therefore hypothesized catumaxomab to influence DAMP sensing immune cells and an activation of an inflammasome. To further probe for a mechanism of liver injury the potential interplay of neutrophils, Kupffer cells and other hepatic accessory cells (NK/NKT and DC) cells was investigated by histopathology.

### Histopathology and immunohistochemistry studies in ALF

**Figure 3:**
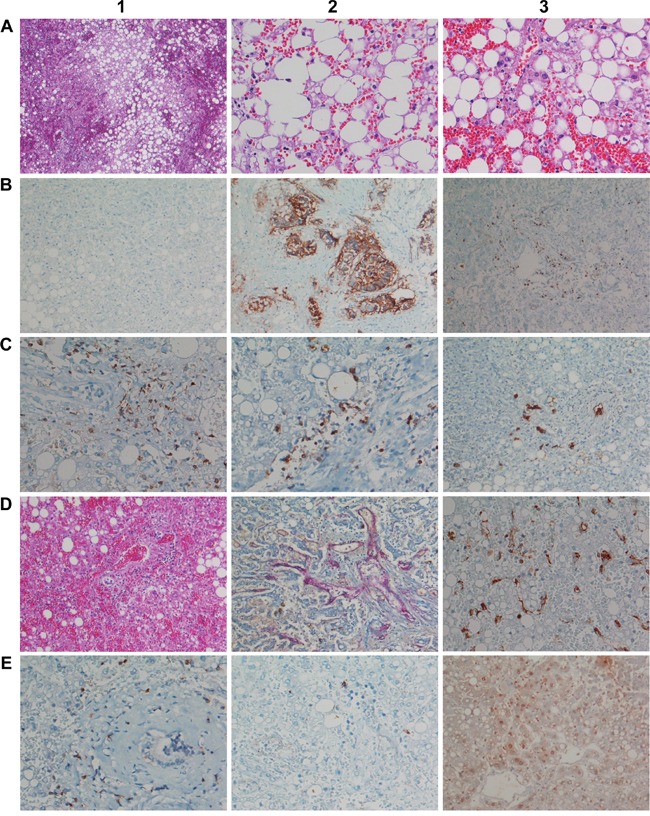
Histopathology and immunohistochemistry of liver tissue from a fulminant fatal case of acute liver failure after a single infusion of 10μg Catumaxomab **A1.** (H&E) Overview of congestive hepatopathy and high grade steatosis (x 50). **A2.** (H&E) Severe macrovesicular steatosis with marked ballooning degeneration (x 200). **A3.** (H&E) Acute hepatic necrosis with extended cellular debris and nuclear fragments, isolated granulocytes intermingled among debris, severe hemorrhage and collapsed central vein. No apoptotic bodies, clear signs of cytolytic damage (x 200). **B1.** Hepatocytes are EpCAM negative; a direct molecular interaction between catumaxomab with hepatocytes is unlikely (x100). **B2.** The lung tumor is EpCAM positive and therefore a bona fide target of catumxaomab; no evidence for T-cell infiltrates and/or antibody dependent cellular toxicity of the tumor (x100). **B3.** Infiltration of the portal tract by CD3 positive lymphocytes (x100). **C1.** Marked portal-periportal infiltration of CD4 positive T-cell (x200). **C2.** Marked portal-periportal infiltration of CD8 positive T-cell concentrated at the portal-hepatocellular interface (x200). **C3.** Immunohistochemistry of cytokeratin 7. Evidence for focal ductular cholangiocellular proliferates within initial septal extension of the portal tract possibly due to a preexisting lesion (x100). **D1.** (H&E) Marked hemorrhage extended to the portal tract; severe hepatocellular damage with small trabecular cell remnants; increased T-cell margination at remnants of the sinusoids; portal infiltration of lymphocytes (x100). **D2.** Staining of portal-periportal vascular structures for CD34. Significant parenchyma damage and destruction results in frequently ruptured sinusoids; the perisinusoidal space of Disse is extensively dilated and blood filled as demonstrated by staining for CD34 of the vascular bed. Small remnants of trabeculae are seen (x100). **D3.** Immunohistochemistry of CD68 reveals activated Kupffer cells (swelling); phagocytosis of cell debris. Besides, signs of sinusoidal rupture and collapse (x200). **E1.** Immunohistochemistry of Granzyme B. Positive lymphocytes are seen and surrounded by a dust of exocytosed granula, particularly at the border of the portal tract (x 200). **E2.** Immunohistochemistry of TIA1; basically no cytoplasmic expression (200x). **E3.** Immunohistochemistry of High mobility group box chromosomal protein 1. Note the significant cytosolic staining of HMGB1 by centrilobular damaged hepatocytes.

In pursuit of mechanisms histological studies were carried out (Figure 3) and next to H&E staining immunohistochemistry involved the T-cell marker CD3, CD4, CD8, the T-cell intracellular and granule-associated protein antigen-1 (TIA-1), perforin and granzyme B, caspase 3, the endothelial /sinusoidal cell markers CD31, CD34, the macrophage marker CD68, the biliary epithelium marker cytokeratins 7 and 20 and HMGB1 to evidence expression of DAMP molecules by harmed hepatocytes.

Microscopic examination revealed a massively congested liver hallmarked by diffuse hemorrhage, sinusoidal degeneration and severe macrovesicular steatosis with signs of ballooning degeneration (Figure 3, A1-A3). At the higher magnification acute hepatic necrosis with fresh cellular debris, nuclear fragments and scarcely intermingled granulocytes between residual lipid droplets were seen (Figure 3, A2, A3). Central veins appeared collapsed while in the periportal zone shedding of cytoplasm at remnants of hepatocellular trabeculae was suspected. Importantly, hepatocytes were negative for EpCAM; thus, a direct molecular interaction of catumaxomab with liver cells was excluded (Figure 3, B1). However, the lung tumor of this patient was EpCAM positive (Figure 3, B2) though no evidence for antibody dependent cellular toxicity (ADCC), tumor lysis and phagocytosis by macrophages was obtained to possibly suggest a regulatory T cell-mediated suppression of tumor clearance.

A prominent finding was the marked lymphocytic infiltration of portal tracts (Figure 3, D1) thus evidencing acute hepatic inflammation. Immunohistochemistry revealed the lymphocytes to be CD3, CD4 & CD8 positive T-cell (Figure 3 B3, C1, C2). The finding that CD3 positive lymphocytes surrounded bile ducts indicates catumaxomab binding to EpCAM positive bile duct epithelium even though their intraductual infiltration was barely seen (Figure 3, C1). When compared to hepatocytes the bile duct epithelium was less harmed, i.e. edema and occasionally desquamation of cells; nonetheless, due to the severe hepatitis an acute intrahepatic cholestasis was suspected with serum bilirubin, alkaline phosphatase and γGT laboratory values being significantly elevated in this patient (Table 1). Staining for CK7 revealed focal periportal bile duct proliferation (Figure 3, G3) possibly due to a preexisting lesion.

Staining of CD68 revealed marked Kupffer cell activation (Figure 3, D3) and catumaxomab's ability to interact with FC fragment sensing macrophages likely contributed to their activation. The local cytokine release mediated by activated Kupffer- and sinusoidal endothelial cells must have critically sensitized hepatocytes for lysis. Overall, increased T-cell margination at remnants of the sinusoids was observed (Figure 3, G3, D1). Indeed, the extraordinary change in serum ALT, AST, LDH and cholesterol is testimony to a massive acute cytolytic hepatitis with cellular and plasma membrane constituents draining into systemic circulation (Table 1, Figure 3, A3).

Some of the lymphocytic infiltrates were positive for granzyme B and surrounded by a dust of exocytosed granula, especially at the interphase (Figure 3, E1). Consequently an up-regulation of cell-death inducing enzymes is seen. Conversely, cytoplasmic expression of TIA-1 was minimal (Figure 3, E2); note, depletion of TIA-1 was shown to promote cell proliferation [10]. Specifically, this RNA/DNA binding protein participates in the transcriptional control of pro-inflammatory cytokines; its minimal expression can be considered as an adaptive response to support cell survival. Additional evidence for an immune mediated acute liver failure stems from HMGB1 immunohistochemistry with its increased hepatocellular cytosolic expression (Figure 3, E3). This protein was repeatedly shown to play a crucial role in the host response to sterile inflammation; its inhibition attenuates damage induced by erroneous programmed innate immune cells.

For comparison, a 65 year old male patient diagnosed with CRC stage IV according to UICC with multiple lung and mediastinal lymph node metastases as well as metastatic infiltration of the hypophysis but no liver metastasis was studied. Therapy involved surgical removal, i.e. deep anterior rectal resection with ascendostomy followed by adjuvant chemotherapy initially with capecitabine in the year 2008 and subsequently with bevacizumab, capecitabine and oxaliplatin in 2009. The 2nd line therapy consisted of bevacizumab, capecitabine and irinotecan in 2010. The 3rd and 4th line therapy comprised lenalidomid and tegafur-uracil in the years 2010 and 2011, and the 5th line therapy was based on mitomycin C and 5-FU also given in 2011.

This patient participated in the phase I trial and received eleven catumaxomab infusions at a dose of 7μg per infusion during the period January 23rd until April 2nd, 2012 [2]. The treatment was reasonable well tolerated; nonetheless the patient succumbed to his cancer 6 weeks after completion of the treatment cycle.

**Figure 4:**
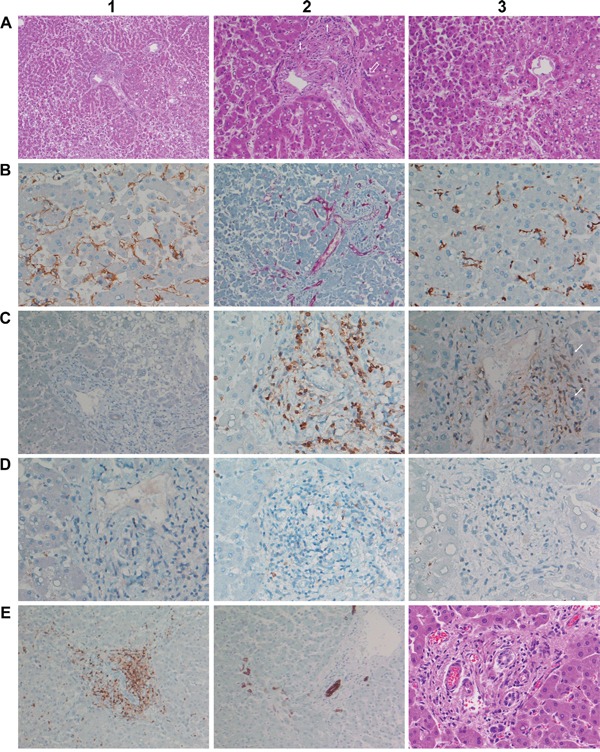
Histopathology and immunohistochemistry of liver tissue from a male patient after 11 consecutive infusions of 7μg Catumaxomab **A1.** (H&E) Moderately altered liver microarchitecture. Signs of low grade reticular fibrosis and partially fragmented trabeculae. Hepatocytes appeared small and slightly basophilic. Note the primarily centrolobular macrovesicular steatosis of about 10 to 20% (x50). **A2.** (H&E) Same image as before but at higher magnification (x100). Moderate portal fibrogenesis with initial septal extensions; partial destruction of the limiting plate and ongoing interface-hepatitis. Focal aggregates of inflammatory cells (open arrow) and bile duct regenerates (small arrows) at the portal edge. Bilayered trabeculae and increased liver cell polyploidization as well as binuclear hepatocytes. Intranuclear glycogen inclusions of hepatocytes. **A3.** (H&E) Pronounced centrolobular macrovesicular steatosis. Signs of enhanced sinusoidal margination of lymphocytes (x100). **B1.** Immunohistochemistry of CD31. Some of the sinusoidal endothelial cells appear enlarged with signs of activation. The counterstain with hematoxylin highlights the frequently bilayered trabeculae (x200). **B2.** Immunohistochemistry of CD34. Hepatic artery and portal vein branches are CD34 positive while sinusoids were CD34 negative. The stain revealed a slightly extended periportal vascular network (x100). **B3.** Immunohistochemistry of CD68. Resident macrophages were moderately activated and enlarged. T-cell margination in proximity to activated Kupffer cells (x200). **C1.** Immunohistochemistry of EpCAM. Hepatocytes were negative for EpCAM. Note the faint membranous EpCAM staining of the bile duct epithelium (x200). **C2.** Immunohistochemistry of CD3. Marked portal T-cell infiltrates. Also visible is the sinusoidal margination of CD3 positive lymphocytes in close proximity to Kupffer cells at the adjacent liver parenchyma (left side, x200). **C3.** Immunohistochemistry of CD4. Marked portal T-cell infiltrates. Bile ducts are rarely infiltrated by lymphocytes; occasionally moderate swelling of ductual epithelium was observed (x200). **D1.** Immunohistochemistry of CD8. Unlike the case of fatal ALF (see Figure 3 C2) only very few lymphocytes are CD8 positive (x200). **D2.** Immunohistochemistry of Granzyme B. Some of the T-lymphocytes are positive and are surrounded by a delicate dust of exocytosed granula, especially at the interphase (x200). **D3.** Immunohistochemistry of TIA1; basically there is no cytoplasmic expression (x200). **E1.** Immunohistochemistry of CD3. T-cell infiltrates particularly at the periportal interphase and the septal extensions; the bile duct is surrounded by T-cells without considerable ductal infiltration (x100). **E2.** Immunohistochemistry of CK7. Bile duct proliferations at the edge of the portal tract and within a septal extension (x100). **E3.** (H&E) An overview of slight interface-hepatitis (x200).

Histopathology revealed the liver microarchitecture to be moderately altered (Figure 4, A1). The trabeculae appeared partly fragmented and dissociated into single cells and a low grade reticular fibrosis is suspected (Figure 4, A1). Frequently, hepatocytes appeared small and slightly basophilic to indicate immature regenerating hepatocytes while part of the trabeculae appeared bi-layered with increased liver cell polyploidization; additionally binuclear hepatocytes were recurrently observed to document increased liver regeneration. Besides, a primarily centrolobular macrovesicular steatosis of 10 to 20% and intranuclear glycogen inclusion of hepatocytes at the periportal zone was seen (Figure 4, A1-3). Some of the sinusoidal endothelial cells and the resident macrophages were moderately activated and enlarged; others presented their typical clinging appearance (Figure 4, B1, B3).

As seen with the ALF case an increased T-cell margination at activated Kupffer cells and/or endothelial cells was observed (Figure 4 B1, B3, C2) 6 weeks after the last catumxomab infusion to possibly suggest catumaxomab induced programming of memory T-cells. Alike, hepatocytes of this male patient were negative for EpCAM. Therefore, a direct interaction of catumaxomab with liver cells was excluded (Figure 4, C1) and the observed sinusoidal margination of T-cells must have been endorsed by the antibody through its binding specificities for Kupffer and T-cells (Figure 4 B1, B3, C2, D1). Moreover, marked portal T-cell infiltrates (Figure 4, C2, C3) though less CD8 positive T-cells (Figure 4, D1) were seen when compared with the ALF case described above and some of the T-lymphocytes were positive for granzyme B and surrounded by a delicate dust of exocytosed granula, especially at the interphase (Figure 4, D2) but were basically negative for T1A (Figure 4, D3). As shown in Figure 4 A2 partial destruction of the limiting plate as well as portal tract fibrogenesis with initial septal extensions was observed. Moreover, T-cell infiltrates particularly at the periportal interphase with focal active inflammatory cell aggregates are notable findings (Figure 4, D1, A2) and the septal extensions of portal tracts with hepatocellular collapse and initial fibrosis were also infiltrated by T-cells (Figure 4, D1).

Note, independent studies revealed bile duct epithelium to be EpCAM positive and therefore constitutes a bona fide target of catumaxomab. In the present study densely packed lymphocytic infiltrates surrounding the bile ducts were observed (Figure 4, D1, D3); however, the lymphocytes rarely infiltrated the ducts itself and the ductual epithelium was mostly intact even though some displayed moderate swelling (Figure 4, C3). Notwithstanding, newly formed bile duct proliferations were seen at the edge of the portal tract and within septal extensions (Figure 4, E2, E3, A2) while staining for the CD34 antigen informed on a slightly extended periportal vascular network (Figure 4, B2) in response to hepatocellular destruction at the limiting plate. Taken collectively, this patient presented pronounced portal-periportal inflammatory cell infiltrates fulfilling the criteria of an interface-hepatitis possible as a result of catumaxomab treatment.

A striking similarity amongst both cases is the pronounced sinusoidal T-cell margination along Kupffer cells to suggest the programing of memory T-cell and T-cell mediated lysis of hepatocytes even though hepatocytes do not express EpCAM.

## DISCUSSION

### Pathogenesis of catumaxomab induced liver injury - towards a general hypothesis –

**Figure 5:**
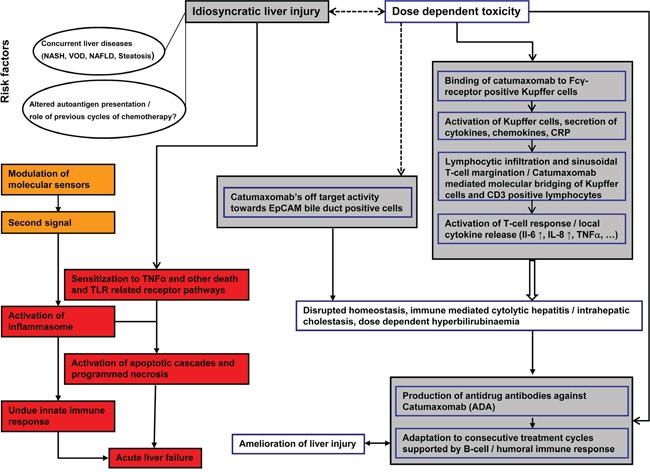
Possible mechanism of catumaxomab induced immune-mediated liver injury

Based on the performed histopathology and clinical observations obtained during a phase I dose escalating trial a mechanism for catumaxomab's liver liability can be hypothesized (Figure 5). Specifically, upon drug administration all patients developed dose dependent hepatitis of different grades with significant serum elevations in ALT, AST, bilirubin and CRP. Most patients experienced chills and pyrexia while plasma cytokine profiling evidenced IL-6 and IL-8 to be significantly increased with peak concentrations at 6 to 9 hours after the start of the infusion. However, no significant change in absolute and relative immune cell count was observed and IFN-γ and TNF-α became undetectable or were very low after repeated treatment [2]. Some patient also developed significant increases in INR (mainly CTCAE Grade 1) to further document liver injury.

Not unexpectedly consecutive catumaxomab infusions induced neutralizing anti-drug antibody (ADA) responses and patients with increased ADA titers were protected, at least in part from liver injury. Owing to its molecular functions catumaxomab binds via its Fc-fragment to FcγR-positive Kupffer cells to increase their activity and to stimulate macrophage dependent CRP, chemokine and cytokine release. The increase in local CRP concentration enhances phagocytosis by Kupffer cells [11] but also stimulates lymphocytic activation and hepatic infiltration as part of the innate immune response. The finding of sinusoidal T-cell margination in close proximity to Kupffer cells is of uttermost importance; it resulted from the binding specificities of the antibody for CD3 positive T-cell and Kupffer cells. Such an antibody mediated “molecular bridging” between T-cells and hepatic macrophages induced a T-cell response and exacerbated local cytokine release and T-cell mediated cytotoxicity. The findings that one patient who succumbed to his cancer 6 weeks after completion of 11 consecutive treatment cycles still presented sinusoidal T-cell margination at activated Kupffer cells may suggest programming of memory T-cells in response to catumaxomab treatment. Importantly, the immune-mediated liver injury induced by catumaxomab does not require hepatocytes to express EpCAM and as seen in the fatal ALF case involved the granzyme B cell death pathway. Moreover, histopathology revealed densely packed lymphocytic infiltrates surrounding bile ducts, however rarely infiltrated bile ducts. Therefore, it remains to be determined whether catumaxomab binding to EpCAM positive bile duct cells contributed to liver injury. Adding to complexity is the significant up-regulation of the DAMP molecule HMGB1 in addition to the cytokines TNFα, IL-6 and IL-8 to counteract the homeostatic response during organ repair.

A summary of the possible mechanism of catumaxomab induced liver injury is depicted in Figure 5. It should be noted that an inflammasome constitutes an intracellular machinery for the maturation of IL-1 and IL-18 [12] with HMGB1 functioning as an “alarm signal” and potent proinflammatory cytokine in multiple signaling pathways [13]. In the present study a highly significant induction of serum HMGB1 was observed and included dose dependent increases for a range of cytokines; however the cytokines IL-1 and IL-18 were not assayed. Hence, an inflammasome mediated mechanism of ALF remains indecisive even though sterile inflammation as a cause of ALF remains unquestionable.

### Paracetamol - an additional culprit in ALF?

In line with the clinical protocol several analgesic and anti-inflammatory medications were foreseen and included 2 grams of paracetamol given intravenously within 6 hours, i.e. 1 gram at the beginning and 1 gram at the end of the catumaxomab infusion. There are reported cases of acetaminophen induced ALF at therapeutic doses [14] and there is experimental evidence for paracetamol to induce the synthesis and release of DAMP molecules into systemic circulation in addition to caspase cleavage of cytokeratin-18 (K18), i.e. an intermediate filament protein expressed by epithelial cells to function in cell structure and integrity. Indeed, DAMPs and K18 serum markers were uniquely associated with systemic immune cell activation but not necessarily influx of inflammatory cells into damaged liver parenchyma as defined by histopathology [15]. Furthermore, paracetamol-induced hepatotoxicity in mice is Toll like receptor 9 and Nalp3 inflammasome dependent [16, 17]. Moreover, cytokines repress the metabolic competence of the liver and activity of the cytochrome P450 monooxygenases required for the detoxification of drugs as was repeatedly observed in patients with SIRS [18, 19]. It is tempting to speculate that paracetamol i.v. administration contributed to DAMP production and associated inflammation thus contributing to ALF.

### Hepatotoxicity with targeted therapies

Apart from paracetamol as a possible culprit bystander our study exemplifies the risk for liver injury by modulating the immune system with molecularly targeted therapies. In the present study extraordinarily small amounts of the antibody were given over a 6 hour infusion, however changing the route of administration caused dose dependent hepatitis even though liver cells do not express EpCAM, i.e. the principal antigen required for the molecular interaction with catumaxomab. It should be noted that average Cmax serum concentrations of about 560 pg/ml were observed in previously performed clinical trials after i.p. administration of 150 mg of catumaxomab and a similar Cmax of 624 pg/ml was determined in the patient with fulminant fatal acute liver failure reported in the present study. Importantly, the liver is a major site for T-cell clearance and the removal of apoptotic as well as non-apoptotic but activated CD8^+^ T cells [20]. Given that all T cells express CD3 an anti-CD3 activation of effector T cells by catumaxomab can be assumed leading to non-specific killing of hepatocytes. Moreover, variability of T-cell CD3 membrane expression influences the capacity for T cell activation and may determine the risk for hepatitis among individual patients [21]. Besides, catumaxomab functions in a non-MHC restricted manner. Therefore, the observed cytolytic hepatitis would not require hepatocytes to express EpCAM but involves the combined activity of cytokines, granzyme B and perforin.

Alike, a recent report highlights the risk for hepatotoxicity for new combination therapies [22]. Specifically, in a phase 1 clinical trial the BRAF inhibitor Vemurafenib and the cytotoxic T-lymphocyte–associated antigen 4 blocking antibody Ipilimumab was given in combination. Although both agents obtained regulatory approval and differ distinctively in their mode of action the combined administration induced hepatic adverse events. Notably, hepatic reactions with Ipilimumab treatment alone are considered to be uncommon, nonetheless guidance for the management of liver injury is available [23, 24].

### Therapeutic strategies in sterile inflammation of the liver

The development of novel therapies for the treatment of sterile inflammation of the liver is the subject of intense research and inhibition of transmission signal 1 and/or signal 2 or its downstream partners may prevent the formation of an active inflammasome [25, 26].

Specifically, HMGB1 may interact with a number of receptors including RAGE and TLRs to promote inflammation [13] and inhibition of HMGB1 activity is an important strategy to alleviate drug induced sterile inflammation.

Immunomodulatory drugs regulate HMGB1 release from activated human monocytes [27]. It is of considerable importance that a previously performed clinical trial evidenced 40 mg of dexamethasone to be effective in ameliorating catumaxomab induced hepatitis [28]. Apart from serum HMGB1 elevated CRP values may serve as a biomarker to identify patients at risk for ALF. Patients with macrovesicular steatosis / NASH are at higher risk for serious liver injury. Hence, uttermost care should be taken with such patients. Avoidance of paracetamol and the timely monitoring for serum markers of inflammation including HMGB1 and pro-inflammatory cytokines is recommended.

## MATERIALS AND METHODS

The patients' characteristics are given in supplementary Table S1 [2]. Blood samples were analyzed for albumin, ALT, AST, γGT, AP, LDH and CRP using commercially available test kits and the cytokines IL-2, IL-4, IL-6, IL-8, IL-10, IL-12, IFN-γ, TNF-α, and GM-CSF were measured by flow cytometry using the commercially available CBA Flex Assay kits (Becton Dickinson).

Histopathology involved H&E staining and immunohistochemistry using the following procedures:

1 μm thick sections were deparaffinised and rehydrated through a descending alcohol series followed by a 4 min washing step in distilled H2O. Subsequently, antigen retrieval was performed in citrate buffer (pH 6) in a water bath at 98°C for 30 minutes. The ZytoChem-Plus HRP Polymer-Kit of Zytomed Systems, Germany was used for immunohistochemistry. The slides were rinsed with distilled H2O, and after a 5 min incubation step in tris-buffered saline (washing buffer) endogenous peroxidase activity was blocked with 3% peroxidase blocking reagent (Merck, Germany) for 5 min followed by a second washing step. Thereafter, the sections were blocked for 5 min with protein-block serum free reagent (ZytoChem-Plus HRP Polymer-Kit, reagent 1) and incubated with primary antibodies for 60 min (see supplementary Table S2).

The bound primary antibodies or bridging antibodies were incubated with labelled polymer HRP Anti-Rabbit or anti-mouse secondary antibody (ZytoChem-Plus HRP Polymer-Kit, reagent 2) for 20 minutes. Subsequently, the reaction was developed and visualized with reagent 3 of the ZytoChem-Plus HRP Polymer-Kit and by placing the slides in a moist chamber at room temperature and allowing for an incubation time of 30 min.

Finally, the sections were counterstained with Haematoxylin for 5 min, washed under running warm tap water for 10 minutes and dehydrated in a cabinet at 60°C for 20 minutes, coverslipped and examined under a light microscope.

### Statistical analysis of the liver function data

Statistical significance testing of liver function tests involved the unpaired, non-parametric Mann-Whitney test with two tails and in the case of cytokines the unpaired t-test with Welch correction using the GraphPad software Prism version 6.0, USA. Data are given as mean and standard deviation.

## CONCLUSION

Owing to catumaxomab's molecular function an erroneous programming of cytotoxic T-lymphocytes and Kupffer cells against EpCAM-negative hepatocytes is hypothesized. The local release of cytokines exacerbates an immune mediated hepatotoxicity and in conjunction with the release of DAMP molecules sterile inflammation is sustained. The observed fulminant fatal liver failure observed in a stage IV CRC patient with multiple lung metastases is the result of a foudroyant disintegration of liver cells (fulminant cytolytic hepatitis) and clinicians should be cautious when changing the route of administration of such medications.

## SUPPLEMENTARY TABLES







## Abbreviations

5-FU: 5-fluorouracil
ADCC: Antibody-dependent cell-mediated cytotoxicity
ALAT: Alanine Aminotransferase
ALF: Acute liver failure
ADA: Anti-drug-antibodies
AP: Alkaline phosphatase
aPTT: activated partial thromboplastin time
ASAT: Aspartate Aminotransferase
ATIII: Antithrombin III
BE(B): Base excess in blood
BEecf: Base excess in extracellular fluid
CA19-9: Carbohydrate antigen 19-9
CEA: Carcinoembryonic antigen
CK: creatine kinase
CRC: Colorectal cancer
CRP: C-reactive protein
CTCAE: Common Terminology Criteria for Adverse Events
DAMPs: damage associated molecular pattern molecules
FA: Folinic acid
Gamma-GT: Gamma-glutamyltransferase
GM-CSF: Granulocytemacrophage colony stimulating factor
HCO3: Bicarbonate
HMGB1: high-mobility group box-1 protein
INR: International normalized ratio
LDH: Lactate dehydrogenase
NSAID: Nonsteroidal anti-inflammatory drug
NT-pro-BNP: N-terminal of the prohormone brain natriuretic peptide
pCO2: Partial pressure of carbon dioxide
pO2: Partial pressure of oxygen
PTZ: Prothrombin time
SIRS: Systemic Inflammatory Response Syndrome (SIRS)
SO2: Oxygen Saturation
TCO2: Total carbon dioxide content of blood
UICC: International Union Against Cancer
