# Supplementary material for: Immune-mediated liver injury of the cancer therapeutic antibody catumaxomab targeting EpCAM, CD3 and Fcγ receptors
Source: Oncotarget. 2016 Apr 4;7(19):28059–74. doi: 10.18632/oncotarget.8574 (PMC5053709; doi:10.18632/oncotarget.8574)
Supplement: Supplementary file 1 [file oncotarget-07-28059-s001.pdf]

Immune-mediated liver injury of the cancer therapeutic antibody catumaxomab targeting EpCAM, CD3 and Fcγ receptors

SUPPLEMENTARY TABLES

Supplementary Table S1: Patients' characteristics

See Supplementary File 1

Supplementary Table S2: List of antibodies used in immunohistochemistry

See Supplementary File 2
